# A Non-Invasive Approach to Intracellular Measurement in Solar Lentigo: Investigating Mitochondrial Dysfunction and Senescence Mechanisms Associated with Excessive Melanin Deposition †

**DOI:** 10.3390/ijms262210918

**Published:** 2025-11-11

**Authors:** Alif Meem Nurani, Takako Shibata, Daigo Inoue

**Affiliations:** MIRAI Technology Institute, Shiseido Co., Ltd., 1-2-11, Takashima, Nishi-ku, Yokohama 220-0011, Japan; alif.nurani@shiseido.com (A.M.N.); takako.shibata@shiseido.com (T.S.)

**Keywords:** solar lentigo, FLIM (fluorescence lifetime microscopy), senescence, melanin, OXPHOS (oxidative phosphorylation)

## Abstract

Solar lentigo is a significant dermatological concern affecting individuals of different genders and ethnicities. Its pathogenesis is primarily attributed to chronic ultraviolet (UV) exposure, increased melanogenesis, and disrupted epidermal turnover, leading to the development of hyperpigmented lesions. A major challenge in solar lentigo research is acquiring viable skin tissue, which is crucial for understanding the dynamics of the cellular microenvironment. In the present study, we sought to establish a non-invasive in vivo measurement technique to visualize cellular dynamics associated with solar lentigo. Utilizing fluorescence lifetime imaging microscopy (FLIM), we quantified the decay of NAD(P)H fluorescence lifetime and observed a reduction in oxidative phosphorylation (OXPHOS) activity in solar lentigo lesions compared to adjacent non-lesional skin. To determine whether the observed reduction in OXPHOS activity was due to excessive melanin accumulation in keratinocytes, we developed a melanin deposition model and examined the pleiotropic alterations occurring in keratinocytes following the phagocytosis of excessive melanin. Our findings indicate that excessive melanin deposition downregulates OXPHOS in differentiating keratinocytes and induces senescence-associated phenotypes characterized by perturbed cell cycle progression, increased cell size and aneuploidy, and the secretion of inflammatory mediators in proliferating keratinocytes. Collectively, our results implicate a solar lentigo-specific senescence mechanism driven by excessive melanin accumulation in keratinocytes, providing new insights about the intrinsic modulators of the pathological condition.

## 1. Introduction

Solar lentigo represents one of the most prevalent skin concerns associated with photoaging across diverse ethnicities and genders, manifesting as the earliest and most prominent sign due to its conspicuous phenotype. These lesions typically appear as unevenly shaped, hyperpigmented patches on areas of the skin frequently exposed to the sun, such as the face and the dorsal side of the hands. The onset of solar lentigo generally occurs in individuals in their 30s, with initial hyperpigmentation primarily observed around the temples and cheeks. Over time, particularly after the age of 40 or 50, these lesions can evolve into various phenotypes characterized by increased quantity, darker pigmentation, and expanded affected areas [1]. Current challenges in the treatment of solar lentigo include the recurrence of lesions even after removal by laser treatment and the limited efficacy of cosmetic ingredients targeting specific molecular pathways. Therefore, fundamental improvement and prevention of solar lentigo require innovative research and development of new approaches to advance the understanding of how its pathology progresses with time.

The hyperpigmentation mechanism underlying solar lentigo can be described by two primary cellular abnormalities: enhanced melanogenesis, marked by the dendritic morphology of melanocytes, and excessive melanin accumulation within basal keratinocytes [2]. Notably, keratinocytes exhibit elevated expression of various inflammatory and hormonal mediators, including GM-CSF, α-MSH, and Endothelin-1, which in turn stimulate the expression of melanogenesis-related factors such as TRP1, MITF, and Tyrosinase in melanocytes, thereby promoting melanogenesis [3,4]. Conversely, basal keratinocytes excessively accumulate melanin derived from melanocytes in the perinuclear region [5]. This process, which leads to the formation of hyperpigmented lesions, is primarily attributed to chronic UV exposure; however, the specific determinants that distinguish the pathological fates of lesional areas from adjacent non-lesional areas, despite their shared history of UV exposure, remain elusive. Prior research has indicated that reduced expression of E-cadherin and Notch1—crucial for the differentiation of epidermal keratinocytes—contributes to the deteriorating microenvironment of solar lentigo through abnormal keratinocyte differentiation, chronic inflammation, and persistent activation of melanocyte-stimulating factors [6,7]. Consequently, in addition to UV exposure as an exogenous factor, it is imperative to consider previously unidentified endogenous factors that may escalate the phenotypes of solar lentigo.

However, obtaining skin samples containing solar lentigo from cosmetic procedures or deceased individuals poses significant challenges, hindering progress in addressing these issues. To overcome this barrier, there has been a longstanding demand for non-invasive measurement techniques for solar lentigo [8,9]. For instance, optical coherence tomography (OCT) vascular imaging has successfully captured abnormal capillary structures within the dermis of solar lentigo, which may play a critical role in establishing the inflammatory milieu [9]. Additionally, fluorescence lifetime imaging microscopy (FLIM) has elucidated melanin distribution in the skin by assessing the fluorescence lifetimes of endogenous fluorescent molecules, including melanin, NAD(P)H, and certain vitamins [10]. Building upon these foundational studies, we hypothesized that fluorescence lifetime imaging could effectively capture the intracellular microenvironment specific to solar lentigo. Thus, the objectives of this study were to: develop a non-invasive measurement approach to elucidate the cellular dynamics of living solar lentigo lesions, and to identify novel intrinsic modulators contributing to the aggravation of solar lentigo by integrating with cell biological assays.

## 2. Results

### 2.1. Decreased Oxidative Phosphorylation (OXPHOS) Activity in the Epidermis of Solar Lentigo

To investigate the metabolic state of epidermal cells in their “living” state, we utilized fluorescence lifetime tomography to measure NAD(P)H, a key electron-transfer molecule essential for redox reactions. NAD(P)H plays a central role in cellular metabolism and exhibits two distinct fluorescence lifetimes, corresponding to its protein-bound and free conformations. These lifetimes provide critical insights into the redox state associated with the two primary ATP-producing metabolic pathways: oxidative phosphorylation (OXPHOS) in mitochondria and glycolysis in the cytosol. In basal keratinocytes, glycolysis is the dominant metabolic pathway, whereas differentiating keratinocytes depend on OXPHOS as a vital source of ATP required for cell differentiation [11]. By analyzing the protein-binding state of NAD(P)H, we can identify metabolic shifts within these ATP-generating pathways in epidermal cells. The fluorescence lifetime of free NAD(P)H typically ranges from approximately 0.2 to 1.2 ns, while the lifetime of bound NAD(P)H extends from 1 ns to 6.5 ns [12]. These lifetimes are influenced by various cellular factors, including pH, the presence of binding proteins, the structure of binding sites, and the overall NAD(P)H pool [13]. Recent research has demonstrated that the amplitude ratio of bound to free NAD(P)H (i.e., the ratio of long-lived to short-lived fluorescence amplitudes) serves as a robust indicator of the cellular redox state [13,14].

**Figure 1:**
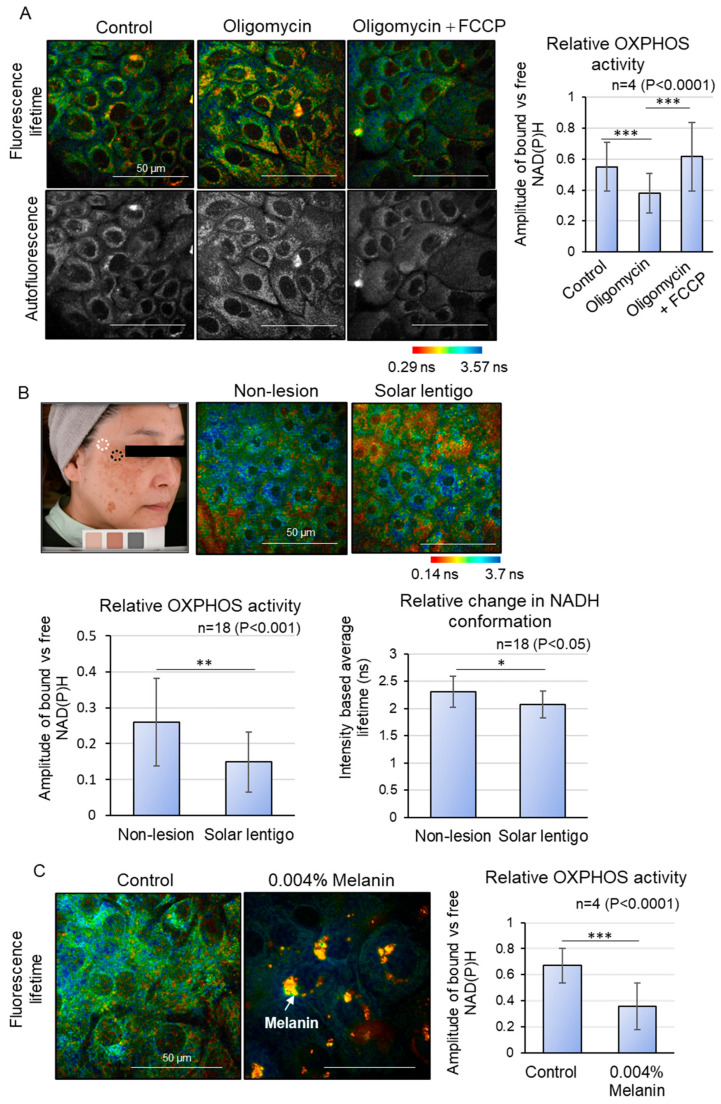
OXPHOS metabolic shift analysis using Fluorescence Lifetime Imaging Microscopy (FLIM). (**A**) FLIM analysis of metabolic shift changes in cultured keratinocytes treated with OXPHOS inhibitor (Oligomycin) and decongestant (FCCP). Oligomycin inhibits OXPHOS activity, which is recovered by FCCP addition. (**B**) FLIM analysis of solar lentigo and peripheral non-lesion areas in vivo. Relative OXPHOS activity and relative change in NADH conformation are downregulated in solar lentigo. (**C**) FLIM analysis of melanin deposited by differentiating keratinocytes in vitro. Relative OXPHOS activity is downregulated upon excessive melanin deposition. Scale bars indicate 50 µm. Statistical significance was calculated using Student’s *t*-test (* *p* < 0.05, ** *p* < 0.001, *** *p* < 0.0001). Sample sizes were n = 4 for (**A**,**C**); n = 18 for (**B**). Error bars indicate mean ± SD.

To validate our approach, we assessed the metabolic state of OXPHOS in keratinocytes differentiated in 1.8 mM CaCl_2_-supplemented medium using an OXPHOS-specific activator (FCCP) and inhibitor (Oligomycin). The results demonstrated that the application of the OXPHOS inhibitor Oligomycin significantly reduced the amplitude of bound versus free NAD(P)H, and subsequent treatment with the OXPHOS activator FCCP restored the effects of Oligomycin, thereby confirming their roles as modulators of the OXPHOS pathway (Figure 1A). In other words, FLIM analysis of cultured keratinocytes indicated that the amplitude ratio of bound to free NAD(P)H is a valid indicator of the state of metabolic shift in OXPHOS activity.

Subsequently, we measured NAD(P)H fluorescence lifetime imaging in live human solar lentigo lesions. (Figure 1B). Due to the difficulty in segregating the fluorescence lifetime decay of free NAD(P)H in the skin’s basal and spinous layers from that of melanin in melanin-deposited basal epidermal cells, we evaluated the granular layer just beneath the stratum corneum (*stratum granulosum* layers 2 and 3), where melanin contribution is minimal and mitochondrial function persists [12]. The intracellular metabolic state was compared between solar lentigo lesions and adjacent non-lesional areas within the same subject. The results indicated that the amplitude of bound versus free NAD(P)H was significantly lower in solar lentigo lesions compared to peripheral non-lesions, suggesting a reduction in OXPHOS activity. This observation was further supported by a decrease in the intensity-based average fluorescence lifetime in solar lentigo lesions, which reflects a shift in NAD(P)H conformation from the protein-bound state to the free state. These findings provide compelling evidence of altered metabolic activity in solar lentigo lesions.

In search of intrinsic modulators that affect OXPHOS activity across differentiating layers, we opted for the role of melanin itself as a physical interference with mitochondrial metabolism, based on the observed spatial proximity between melanin and mitochondria within cells. We hypothesized that excessive melanin accumulation in the basal layer keratinocytes of solar lentigo may eventually impact mitochondrial OXPHOS activity in the differentiating layer. To explore this, we developed a melanin deposition model in which basal keratinocytes were phagocytosed with synthetic melanin to form a perinuclear melanin cap [15]. After 3 days of melanin incorporation, the keratinocytes were supplemented with 1.8 mM CaCl_2_ to induce differentiation for 5 days, and OXPHOS activity was analyzed via FLIM (Figure 1C). The ROI was meticulously selected to exclude pixels containing melanin, which exhibit very short lifetimes during analysis. Notably, the amplitude of bound versus free NAD(P)H in the cytosol was significantly diminished in melanin-accumulated differentiating keratinocytes compared to control differentiating keratinocytes, indicating that the presence of melanin within cells can reduce mitochondrial OXPHOS activity during differentiation (Figure 1B). These findings suggest that reduced OXPHOS activity in the differentiating layer of lesions may be linked to the differentiation abnormalities observed in solar lentigo.

### 2.2. Excessive Melanin Deposition in Keratinocytes Induces Cellular Senescence

**Figure 2:**
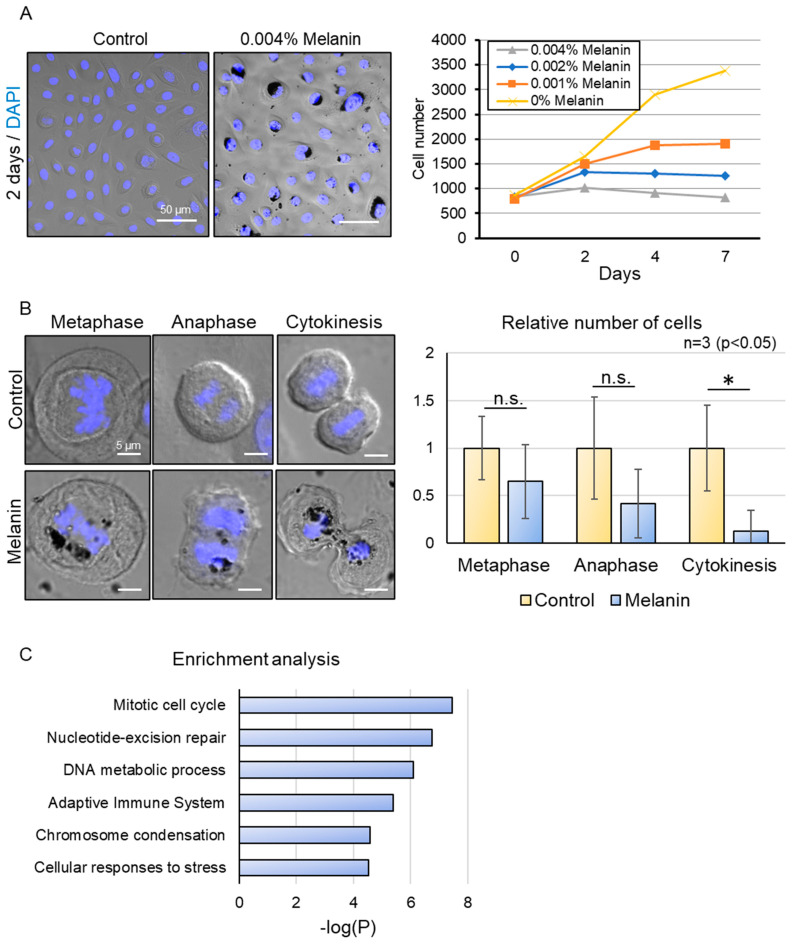
Effect of excessive melanin deposition on the mitotic cell cycle process. (**A**) Representative images of control (0% Melanin) and 0.004% melanin-treated keratinocytes (left). Inhibition of cell proliferation in melanin-deposited keratinocytes in a dose-dependent manner (right). (**B**) Representative images of metaphase, anaphase, and cytokinesis in control and melanin-deposited keratinocytes (left). Decrease in the relative number of cells undergoing cytokinesis in melanin-deposited keratinocytes. (**C**) Enrichment analysis of differentially expressed proteins (2-fold) after LC-MS/MS analysis of melanin-deposited basal keratinocytes relative to control, showing alteration of the cell cycle process and DNA homeostasis. Scale bars indicate 50 µm for (**A**) and 5 µm for (**B**). Statistical significance was calculated using Student’s *t*-test (* *p* < 0.05, n.s. indicates non-significant) for (**B**), and a cut-off value above 4.5 for −log(P) was chosen for (**C**). Sample sizes were n = 3 for (**A**,**B**); n = 4 for (**C**). Error bars indicate mean ± SD.

Until now, we have investigated the microenvironment of solar lentigo and identified a deterioration of mitochondrial oxidative phosphorylation (OXPHOS) metabolism in the differentiating layer. Our findings also suggest that excessive melanin accumulation in keratinocytes may act as a danger signal, indicating metabolic changes associated with solar lentigo. To understand how basal keratinocytes that accumulate melanin can transduce signals to the differentiating layer, we next examined the characteristics of these melanin-accumulating basal cells, specifically whether they exhibit senescence phenotypes. Utilizing the previously described melanin deposition model, we assessed the impact of melanin on keratinocyte proliferation in a dose-dependent manner. While the number of cells gradually increased in the control group, melanin incorporation resulted in cell cycle arrest in a concentration-dependent manner (Figure 2A). To determine how melanin deposition affects the mitotic cell cycle, we carefully observed and quantified the number of cells at each mitotic phase after a 3-day treatment with high-dosage melanin (Figure 2B). Although the reduction in the number of cells in metaphase and anaphase was not statistically significant, a notable decrease in the relative number of cells undergoing cytokinesis was observed in melanin-deposited keratinocytes. Additionally, we observed that due to the proximity of excessive melanin to the nucleus, segregation of sister chromatids was mildly disrupted during anaphase, leading to asymmetrical nuclear division during cytokinesis. To obtain a comprehensive overview of the molecular changes that occur after melanin deposition, we compared the protein compositions with those of the control. About 3590 annotated proteins were found, and GO enrichment analysis was conducted for proteins with a differential expression of over 2-fold (Figure 2C). We confirmed that the mitotic cell cycle process, specifically associated with chromosome segregation, was indeed affected in melanin-deposited keratinocytes, followed by proteins involved in DNA replication and repair. Moreover, the adaptive immune system was activated, and cellular proteins were responding to stress signals. Notably, we identified that the mitochondrial complex IV assembly protein COX7C was significantly downregulated in melanin-deposited keratinocytes, which is consistent with our findings of a reduction in COX7C expression in hyperpigmented tissues [16]. Our results indicate that melanin hyperaccumulation may induce cytotoxic signals that disrupt the nuclear and mitochondrial homeostasis in keratinocytes, contributing to altered mitotic progression and energy metabolism, potentially impacting the basal dividing cells in solar lentigo.

**Figure 3:**
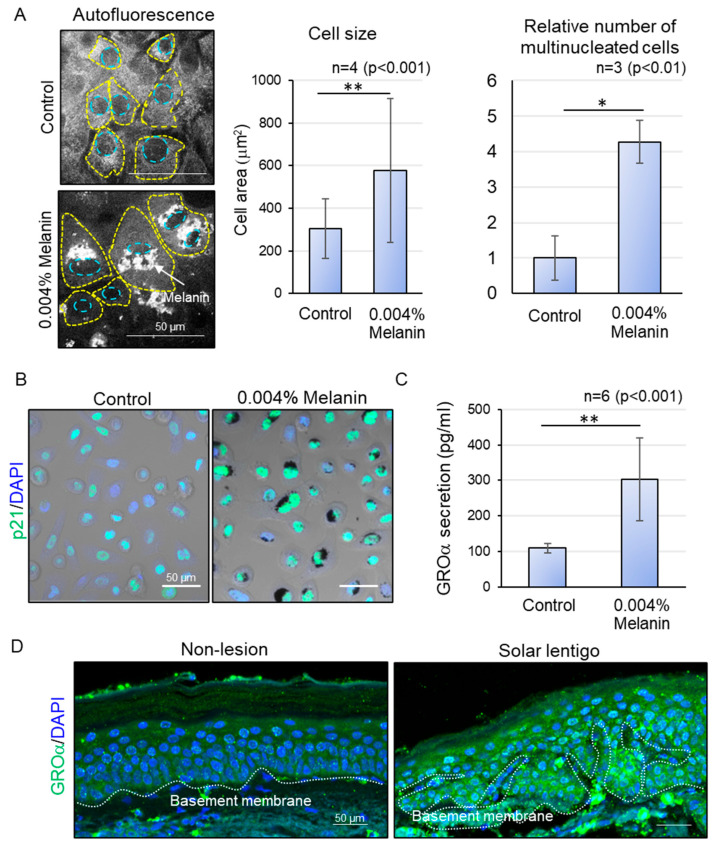
Effect of excessive melanin deposition on cellular senescence. (**A**) Representative autofluorescence images of control and melanin-deposited keratinocytes (left) and an increase in cell size and number of multinucleated cells at 0.004% concentration of melanin (right). (**B**) Increase in nuclear p21 expression in melanin-deposited keratinocytes. (**C**) Increase in GROα secretion in supernatants of melanin-deposited keratinocytes. (**D**) Increased GROα expression in hyperpigmented areas with higher epidermal thickness, compared to peripheral non-lesions in tissue samples from the same donor. Statistical significance was calculated using Student’s *t*-test (* *p* < 0.01, ** *p* <0.001). Sample sizes were n = 3 for (**A**,**B**), n = 6 for (**C**), and n = 5 for (**D**). Scale bars indicate 50 µm. Error bars indicate mean ± SD.

To determine if such subcellular anomalies can lead to the onset of senescence, we subsequently examined the cellular morphology and expression of senescence markers. Melanin-deposited keratinocytes demonstrated a marked enlargement in cell size, as well as a higher proportion of multinucleated cells relative to untreated cells (Figure 3A). Furthermore, we observed the nuclear localization of p21 in melanin-deposited keratinocytes through immunofluorescence staining using confocal laser microscopy. After five days of prolonged melanin accumulation, keratinocytes exhibited a significantly increased nuclear localization of p21 compared to control basal keratinocytes (Figure 3B). These findings collectively indicate that keratinocytes with hyperaccumulated melanin exhibit morphological hallmarks indicative of cellular senescence.

Finally, to explore the possibility that the melanin deposited abnormally dividing keratinocytes can transduce signals to neighboring cells, we assessed the secretion of cellular senescence-associated secretory phenotype (SASP) factors using a cytokine array, the results of which were confirmed with ELISA. The supernatant from melanin-hyperaccumulating keratinocytes demonstrated a significant increase in the secretion of the SASP factor GROα [17] compared to that of control (Figure 3C). GROα is known to be secreted by stressed keratinocytes, which eventually leads to abnormal differentiation and epidermal thickening during the pathogenesis of psoriasis [18]. Interestingly, in hyperpigmented tissues, GROα was also found to be upregulated specifically in hyperpigmented areas with larger epidermal thickness, compared to peripheral non-pigmented regions with thinner epidermis (Figure 3D), suggesting that melanin-induced release of chemokine signals can be effectively transmitted to the upper differentiating layers, disrupting the normal differentiation process and contributing to an abnormal increase in epidermal thickness.

In conclusion, our findings indicate that hyperaccumulation of melanin in basal keratinocytes can trigger a cascade of cellular abnormalities, including aberrant mitotic division, nuclear polyploidy, increased cell size, nuclear localization of the cell cycle regulator p21, and the secretion of senescence-associated secretory phenotype (SASP) factors. Collectively, all these changes are indicators of cellular senescence that may drive the downregulation of OXPHOS metabolism and promote abnormal differentiation in the upper layers of the epidermis. These findings highlight a multifaceted and integrative senescence mechanism that is uniquely associated with the pathogenesis of solar lentigo.

## 3. Discussion

### Integrative Approach of Non-Invasive Measurement and Cell Biology of Intracellular Dynamics Reveals Comprehensive Process of Deterioration of Solar Lentigo

The challenges associated with obtaining fresh samples of solar lentigo have significantly limited the ability to analyze intracellular changes in its pathology in real time. Noninvasive measurement techniques, such as reflectance confocal microscopy (RCM) and optical coherence tomography (OCT angiography), have successfully visualized the tissue-level microstructure of solar lentigo [19]. However, these methods have struggled to provide detailed insights into the dynamic changes occurring at the cellular level. Conversely, studies conducted under limited spatiotemporal conditions have identified distinctive elements and pathways involved in melanogenesis, inflammation, and epidermal turnover. However, they have not offered a comprehensive overview of the life cycle of solar lentigo, including their emergence and progression with age [20,21]. In this study, we employed an integrated approach that combines non-invasive measurement techniques with advanced cell biological methods to elucidate the intrinsic modulators of solar lentigo, specifically focusing on melanin hyperaccumulation. Although our population size is relatively small, one key challenge in recruiting a larger cohort lies in the specific requirements for accurate measurement of solar lentigo using the equipment. For reliable detection via FLIM, the lesion must be located near the temple or cheek region, and the skin must be adequately hydrated. Nevertheless, we believe this pilot study offers valuable insights and serves as a basis for future investigations with larger and more balanced populations. Additionally, we demonstrated a specific cellular senescence process that amplifies the pathology of established solar lentigo. Our results provide critical insights into the specific microenvironmental cues that contribute to the deterioration of solar lentigo lesions in contrast with neighboring non-lesions possessing a similar history of UV exposure.

Skin disorders such as solar keratosis and seborrheic keratosis, which are indicative of dysfunction in epidermal keratinocyte proliferation and differentiation, are thought to involve keratinocyte cellular senescence in their pathogenesis [22,23]. However, the role of keratinocyte cellular senescence in solar lentigo remains unclear. Preliminary studies have indicated that basal keratinocytes undergoing cellular senescence due to UVB irradiation and other factors promote melanin uptake [24], yet the extent to which melanin hyperaccumulation induces cellular senescence in keratinocytes has not been thoroughly investigated. Moreover, although melanin acts as a radical scavenger, offering protection to keratinocytes against UV-induced DNA damage and reduction in oxidative stress [25,26], the physical and long-term impacts of perinuclear melanin within keratinocytes have not been sufficiently explored. Our findings provided new insights into how the excessive accumulation of melanin can itself act as a physical barrier, interfering with chromatin assembly and the function of subcellular organelles, which in turn, activate senescence programs at a chronic level.

A similar phenomenon can be observed in melanin within the retinal pigment epithelium (RPE), which primarily serves a protective role against photodamage; however, with the progression of age, it undergoes oxidative modifications that enhance its photoreactivity while reducing its antioxidant capacity. The inability of melanin to effectively degrade lipofuscin leads to the formation of large melanolipofuscin aggregates, which disrupt the homeostasis of the RPE. Such structural and functional abnormalities in melanin, coupled with the role of RPE mitochondrial dysfunction, have been implicated in the pathogenesis of age-related macular degeneration (AMD) [27]. Similarly, neuromelanin, which accumulates in various neurons, is also known to undergo structural alterations in conjunction with α-synuclein accumulation, contributing to neurodegenerative processes [28]. These examples suggest the potential that prolonged excessive melanin deposition in solar lentigo may result in structural and functional abnormalities, transforming the role of melanin from a UV protector to a contributor to melanin-induced senescence phenotypes.

The ingestion of fine particulate matter (PM2.5) has been shown to trigger cell cycle arrest and promote cellular senescence in keratinocytes, ultimately contributing to the development of skin conditions such as allergies, inflammatory dermatitis, and eczema [29]. While the findings regarding phagocytosed melanin in this study bear similarities to these effects, it is important to emphasize that the use of synthetic melanin in our experiments was specifically chosen to observe dose-dependent changes and the long-term persistence of melanin within cells. Although the use of melanosomes may be more physiologically relevant in the context of normal skin pigmentation, our methodology provides unique insights into the compositional differences in melanin and its adverse properties in pathological conditions. An additional consideration is whether the concentration-dependent effects of melanin accumulation suggest an increased likelihood of cellular senescence in individuals with darker skin tones. While this hypothesis warrants further exploration, preliminary data from our study indicate that the detrimental effects of excessive melanin accumulation are independent of skin type. This suggests that, although different ethnic groups may exhibit varying thresholds for melanin overaccumulation, the long-term consequences of such accumulation in solar lentigo appear to be universally harmful across all skin types. Emerging evidence suggests that in persistent pigment darkening, alterations in both eumelanin and pheomelanin chromophores are associated with the generation of reactive oxygen species (ROS). Studies have demonstrated that the specific composition of melanin, characterized by defined ratios of DHI (35%), DHICA (41%), BZ (20%), and BT (4%), remains consistent irrespective of pigmentation levels. The high DHICA content is suggested to offer antioxidant properties, whereas the ratios of eumelanin to pheomelanin and BZ to BT moieties in pheomelanin are critical determinants of its pro-oxidant potential [30]. These findings suggest that melanin-induced cytotoxicity may be more intricately linked to its molecular composition than to its absolute quantity. Future research should aim to investigate the prolonged effects of different types of melanin on skin homeostasis, with a particular focus on understanding these processes across diverse ethnicities.

The role of melanin pigments has also been implicated in melanin-associated pathologies, such as melanoma. While UVB-induced melanoma primarily arises from direct DNA damage, UVA-induced melanoma is associated with the presence of melanin pigment and oxidative DNA damage within melanocytes [31]. Interestingly, mitochondrial dynamics and energy metabolism exhibit remarkable plasticity in melanoma, adapting to the tumor microenvironment. For instance, in glucose-deprived conditions, tumor cells undergo metabolic reprogramming, favoring elevated oxidative phosphorylation (OXPHOS). Conversely, under hypoxic conditions, melanoma cells predominantly rely on glycolysis, with metabolic preferences influenced by the tumor cells’ proximity to the vasculature [32]. Furthermore, aggressive melanomas exhibit significant upregulation of mitochondrial translation and OXPHOS, underscoring the dynamic nature of energy metabolism in these tumors [33].

Conversely, in melanin-deficient pathologies such as vitiligo, impaired electron transport chain (ETC) activity and reduced ATP production have been observed in melanocytes. Additionally, keratinocytes in perilesional lesions display abnormal mitochondrial ultrastructure, including disorganized cristae, increased mitochondrial size, whereas fibroblasts exhibit signs of premature senescence and elevated oxidative stress [34]. These findings, together with our current study on solar lentigo, highlight the complex and multifaceted role of melanin across diverse physiological and pathological contexts. It emphasizes the need for detailed reviews and further studies to understand how melanin composition and mitochondrial dynamics influence normal skin function and disease development.

## 4. Materials and Methods

### 4.1. Human Study

The study was conducted according to the guidelines of the Declaration of Helsinki and approved by the Shiseido Ethics Committee (Approval No. C10290). Written informed consent was obtained from all 18 participants, comprising 14 women and 4 men, aged between 30 and 69 years, each diagnosed by a dermatologist with prominent solar lentigo lesions on the cheek. Furthermore, additional consent was obtained from participants for the use of photographic images (with eyes covered) in the study figures. Prior to testing, participants cleansed their faces and acclimatized to room conditions for 15 min, with the environment maintained at a temperature of 22 ± 1 °C and relative humidity of 45 ± 5%.

### 4.2. Multiphoton Laser Tomography and FLIM Analysis

In vivo skin measurements and in vitro cell culture FLIM assays were conducted using the MPTcompact and MPTflex systems (JenLab, Berlin, Germany) with excitation wavelengths ranging from 760 to 780 nm and a maximum power output of 10–20 mW. Fluorescence lifetime analysis was performed utilizing Symphotime 64 (ver. 2.4, PicoQuant, Berlin, Germany) and SPCImage software (ver. 8.2, Becker & Hickl, Berlin, Germany). The fluorescence decay curves were fitted using bi- or tri-exponential decay models, as referenced in previous studies [11], following Fourier transformation via the phasor method to assess contributions from mixed species with varying lifetimes. For each FLIM image, we analyzed 3–5 regions of interest (ROI) within the cell cytoplasm. The amplitude ratio of bound NADPH lifetime to free NADPH lifetime (A_bound NADPH_/A_free NADPH_) and intensity-based average lifetime were calculated to assess the metabolic shift.

### 4.3. Preparation of Melanin-Deposited Basal and Melanin-Deposited Differentiating Keratinocytes

Normal human epidermal keratinocytes (Kurabo, Osaka, Japan) were cultured in EpiLife medium (MEPI500CA, Thermo Fisher Scientific, Waltham, MA, USA) supplemented with the HuMedia KG Growth Factor Kit (KK-6150, Kurabo). Synthetic melanin (M0418, Sigma-Aldrich, St. Louis, MI, USA) was prepared by dissolving it in 1× PBS to create a stock solution at a concentration of 1% (*w*/*v*), followed by sonication at room temperature for 2 h. The sonicated melanin was subsequently diluted to various final concentrations (0.001–0.004%) using EpiLife culture medium. The cultured keratinocytes were then incubated with the melanin-containing medium for 24 h. Following incubation, the dishes were washed twice with 1× PBS to eliminate any residual melanin that had not been phagocytosed by the keratinocytes. These melanin-deposited basal cells were then cultured for up to 7 days, depending on the experimental procedure. For the preparation of melanin-deposited differentiating keratinocytes, after 3 days of melanin incorporation, the keratinocytes were supplemented with 1.8 mM CaCl_2_ to induce differentiation for an additional 5 days.

### 4.4. Immunofluorescence of Facial Skin Specimens and Melanin-Deposited Keratinocytes

Facial skin specimens exhibiting hyperpigmented lesions were procured from Obio LLC (El Segundo, CA, USA) and CTIBiotech (Rhone, France) (Approval No. C10485). Paraffin-embedded samples (*n* = 5, comprising two males and three females, aged 79–91 years) were histologically sectioned, followed by immunofluorescence staining to investigate the expression patterns of GROα (12335-1-AP, Proteintech, Rosemont, IL, USA). For immunostaining of keratinocytes with melanin deposition, cells were fixed using 4% paraformaldehyde (PFA) and subsequently stained with p21 Waf1/Cip1 antibody (2947T, Cell Signaling Technology, Danvers, MA, USA) and Hoechst 33258 (Thermo Fisher Scientific). Static images of the immunostained samples were captured using a 20× objective lens on confocal microscopes (LSM700 and LSM880, ver. ZEN 2.0, Carl Zeiss, Oberkochen, Germany) and further analyzed in ImageJ software (ver. 1.54).

### 4.5. Liquid Chromatography-Tandem Mass Spectrometry (LC-MS/MS)

Samples were prepared using the EasyPep™ 96 MS Sample Prep Kit (Thermo Fisher Scientific) as per the manufacturer’s instructions. LC-MS/MS analysis was performed on an Ultimate 3000 RSLCnano system coupled with an Orbitrap Fusion Lumos (Thermo Fisher Scientific), using a nano-HPLC capillary column (ODS, 75 μm i.d. × 120 mm, particle size: 3.0 μm, Nikkyo Technos Co., Ltd., Tokyo, Japan). Peptide separation was achieved with a linear gradient of 2−35% mobile phase B (0.1% formic acid in acetonitrile) over 120 min, with mobile phase A being 0.1% formic acid in water. Data-dependent acquisition mode was used for precursor isolation and sequencing. Raw LC-MS/MS data was analyzed by Proteome Discoverer (version 2.3), and label-free quantitation (LFQ) was performed for each parameter group using unique and razor peptides. Gene ontology (GO) enrichment analysis was conducted with Metascape [35].

### 4.6. Quantification of Cell Viability, Cell Number, and Cell Size

Cell viability was assessed using either the AlamarBlue cell viability reagent (DAL1025, Thermo Fisher Scientific) or Hoechst 33258, with measurements taken using a multi-detection microplate reader (POWERSCAN HT, BioTek, Winooski, VT, USA). To determine the relative cell count, cells were selected based on distinctive nuclear features: those exhibiting condensed chromosomes at metaphase, clearly aligned and segregated sister chromatids at anaphase, and undergoing both cellular and nuclear division during cytokinesis. Cells containing more than one nucleus were classified as multinucleated. All measurements were normalized to the total cell count within the observed range. The cell area was quantified using autofluorescence images analyzed with ImageJ software (ver. 1.54).

### 4.7. Detection of SASP Factors by ELISA

Melanin-deposited keratinocytes were cultured for 5 days without medium replacement. The supernatants were subsequently analyzed using the Human CXCL1/GRO alpha Quantikine ELISA Kit (DGR00B, RD Systems, Tustin, CA, USA). The total secretion levels were normalized to the total viable cell count for each individual sample.

### 4.8. Statistical Analysis

Data are expressed as the mean ± standard deviation. Statistical analyses were conducted using GraphPad Prism (ver. 10.4.1). A *p*-value of less than 0.05 was considered indicative of statistical significance.

## 5. Conclusions

Traditional research methods on solar lentigo have primarily focused on examining selective regulatory factors within the epidermal and dermal layers of the skin. While these studies have provided valuable insights into various aspects, such as fibroblast senescence, abnormal capillary formation, increased nerve fibers, and the presence of melanophages in the dermis, as well as the processes of melanogenesis, melanin transfer, and accumulation in keratinocytes in the epidermis, a significant challenge has persisted. Specifically, it has been immensely challenging to elucidate how these processes collectively contribute to the progression of solar lentigo with advancing age. This study offers a fresh perspective by introducing a novel understanding of the solar lentigo-specific senescence mechanism, which is driven by excessive melanin deposition. This allows us to provide a comprehensive framework for understanding how the interplay and timing of these individual regulatory factors influence the lifecycle of solar lentigo. This integrative approach not only enhances our understanding of the pathological condition but also holds the potential to pave the way for developing more effective strategies to mitigate the age-related deterioration of solar lentigo. Addressing these complex interactions may enable future research to develop targeted interventions that may slow down or even prevent the progression of solar lentigo, ultimately improving the overall skin health and appearance.

## Data Availability

The original contributions presented in this study are included in the article. Further inquiries can be directed to the corresponding author.
